# 406. Incidence and Risk Factors for Hospital-Acquired Blood Stream Infections in Patients Hospitalized with COVID-19 in the University Medical Centre, Lithuania

**DOI:** 10.1093/ofid/ofad500.476

**Published:** 2023-11-27

**Authors:** Ieva Kubiliute, Jurgita Urboniene, Fausta Majauskaite, Birute Zablockiene, Ligita Jancoriene

**Affiliations:** Clinic of Infectious Diseases and Dermatovenerology, Institute of Clinical Medicine, Faculty of Medicine, Vilnius University, Vilnius, Lithuania, Vilnius, Vilniaus Apskritis, Lithuania; Center of Infectious Diseases, Vilnius University Hospital Santaros Klinikos, Vilnius, Lithuania, Vilnius, Vilniaus Apskritis, Lithuania; Clinic of Infectious Diseases and Dermatovenerology, Institute of Clinical Medicine, Faculty of Medicine, Vilnius University, Vilnius, Vilniaus Apskritis, Lithuania; Clinic of Infectious Diseases and Dermatovenerology, Institute of Clinical Medicine, Faculty of Medicine, Vilnius University, Vilnius, Lithuania, Vilnius, Vilniaus Apskritis, Lithuania; Clinic of Infectious Diseases and Dermatovenerology, Institute of Clinical Medicine, Faculty of Medicine, Vilnius University, Vilnius, Lithuania, Vilnius, Vilniaus Apskritis, Lithuania

## Abstract

**Background:**

Bacterial co-infections increase the severity of COVID-19 and are frequent causes of mortality in patients with COVID-19. The study aimed to describe hospital-acquired blood stream infections (BSI) in hospitalized patients with COVID-19 infection.

**Methods:**

Retrospective observational cohort study took place in Vilnius University Hospital Santaros Klinikos (VUH SK), Lithuania. Inclusion criteria were adult patients hospitalized to VUH SK with confirmed COVID-19 infection between March 2020 and May 2021. Depersonalized data were retrieved from electronic medical records. BSI was defined as the growth of a non-skin flora commensal on one or more blood culture (BC). Bacteria belonging to the commensal skin flora (coagulase-negative staphylococci, Micrococcus spp., Propionibacterium spp., Corynebacterium spp.) growing in BC sets are defined as contaminants. Only one BSI episode was counted when several BC sets were positive with the same microorganism for a patient.

**Results:**

We evaluated 2799 adults with COVID-19. Total 2239 BC were taken from 741 patients ≥48 hours from admission. 141 hospital-acquired infections were documented in 101 patients. Main characteristics of patients are shown in Table 1. The most frequent pathogens causing hospital-acquired BSI were Acinetobacter baumanii, Klebsiella pneumoniae, Staphylococcus spp. and Enterococcus faecium (Fig. 1). Atrial fibrillation, obesity, chronic kidney disease, previous stroke, and need of invasive ventilation (IV) were associated with increased risk of gram-negative hospital-acquired BSI and previous stroke, need of IV were associated with increased risk of gram-positive BSI (Fig.2). Patients with hospital-acquired BSI had higher risk of in-hospital mortality within 60 days (HR 1.73 (1.27 – 2.36), p=0.001) (Fig.3).

**Table 1.**
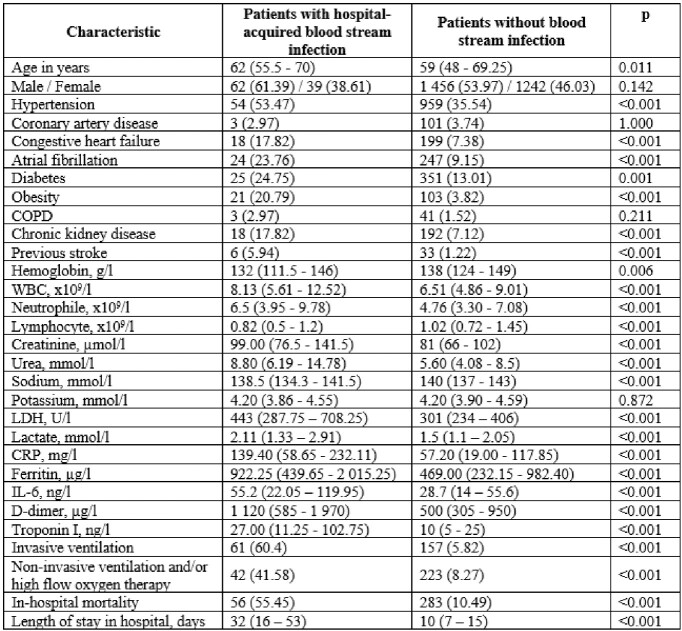
Baseline characteristics of hospitalized patients. Data is presented as median (25%, 75% quartiles) or n (%). Results by chi square test, or Fisher’s exact test for categorical variables and by Mann–Whitney U test for continuous variables. CRP – C-reactive protein; IL-6 – interleukin-6; LDH – lactate dehydrogenase; WBC – white blood cell count; COPD – chronic obstructive lung disease.

**Figure 1.**
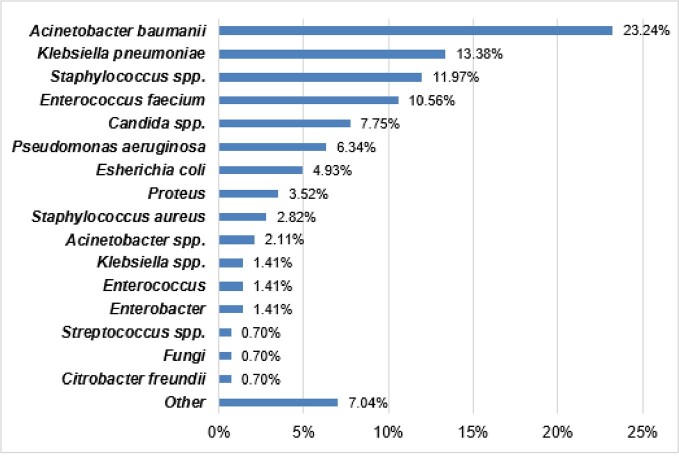
Pathogens of hospital-acquired blood stream infections. Data reported as proportion (%) of the total number of pathogens.

**Figure 2.**
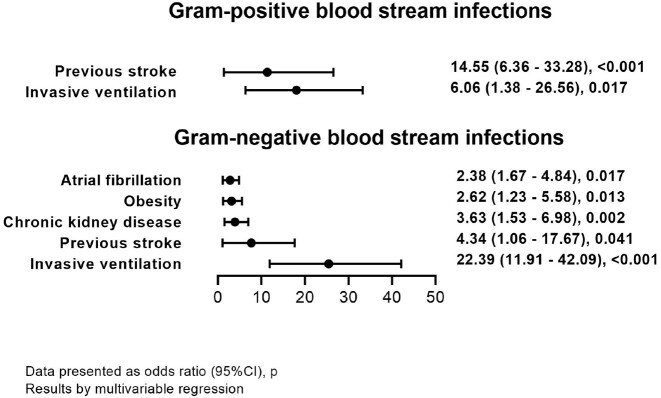
Risk factors associated with hospital-acquired blood stream infections.

**Conclusion:**

Incidence of hospital-acquired blood stream infections in hospitalized patients with COVID-19 was 3.6% which increased the risk for in-hospital mortality by 1.73 times. Atrial fibrillation, obesity, chronic kidney disease, previous stroke, and need of invasive were associated with increased risk of hospital-acquired blood stream infections.

**Figure 3.**
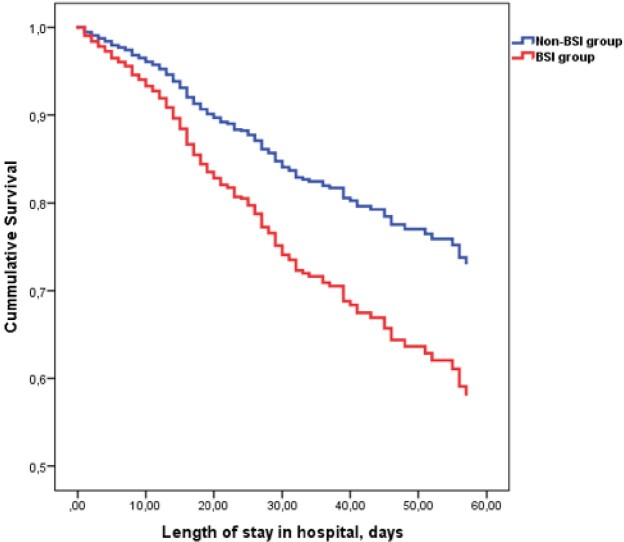
Survival of hospitalized patients with COVID-19 stratified by hospital-acquired blood stream infection group.

Results by Cox regression. BSI - blood stream infection.

**Disclosures:**

**All Authors**: No reported disclosures

